# Intravenous Bone Marrow Mononuclear Cells Transplantation in Aged Mice Increases Transcription of Glucose Transporter 1 and Na^+^/K^+^-ATPase at Hippocampus Followed by Restored Neurological Functions

**DOI:** 10.3389/fnagi.2020.00170

**Published:** 2020-06-11

**Authors:** Yukiko Takeuchi, Yuka Okinaka, Yuko Ogawa, Akie Kikuchi-Taura, Yosky Kataoka, Sheraz Gul, Carsten Claussen, Johannes Boltze, Akihiko Taguchi

**Affiliations:** ^1^Department of Regenerative Medicine Research, Foundation for Biomedical Research and Innovation at Kobe, Hyogo, Japan; ^2^Multi-Modal Microstructure Analysis Unit, RIKEN-JEOL Collaboration Center, RIKEN, Hyogo, Japan; ^3^Laboratory for Cellular Function Imaging, RIKEN Center for Biosystems Dynamics Research, RIKEN, Hyogo, Japan; ^4^Fraunhofer Institute for Molecular Biology and Applied Ecology IME – ScreeningPort, Hamburg, Germany; ^5^Fraunhofer Cluster of Excellence Immune-Mediated Diseases CIMD, Hamburg Site, Hamburg, Germany; ^6^School of Life Sciences, University of Warwick, Coventry, United Kingdom

**Keywords:** bone marrow mononuclear cell, stem cell, glucose transporter, Na^+^/K^+^-ATPase, quantitative PCR

## Abstract

We recently reported that intravenous bone marrow mononuclear cell (BM-MNC) transplantation in stroke improves neurological function through improvement of cerebral metabolism. Cerebral metabolism is known to diminish with aging, and the reduction of metabolism is one of the presumed causes of neurological decline in the elderly. We report herein that transcription of glucose transporters, monocarboxylate transporters, and Na^+^/K^+^-ATPase is downregulated in the hippocampus of aged mice with impaired neurological functions. Intravenous BM-MNC transplantation in aged mice stimulated the transcription of glucose transporter 1 and Na^+^/K^+^-ATPase α1 followed by restoration of neurological function. As glucose transporters and Na^+^/K^+^-ATPases are closely related to cerebral metabolism and neurological function, our data indicate that BM-MNC transplantation in aged mice has the potential to restore neurological function by activating transcription of glucose transporter and Na^+^/K^+^-ATPase. Furthermore, our data indicate that changes in transcription of glucose transporter and Na^+^/K^+^-ATPase could be surrogate biomarkers for age-related neurological impairment as well as quantifying the efficacy of therapies.

## Introduction

Although only contributing to about 2% of the bodyweight, the brain receives approximately 20% of blood flow in the steady state, demonstrating its substantial metabolic demand. Since the brain does not possess relevant energy stores, it is essential that appropriate cerebral blood flow (CBF) occurs, thereby ensuring an uninterrupted supply of nutrients to sustain neuronal metabolism. Neurological impairments in the elderly, including both vascular dementia and Alzheimer’s disease, are known to be associated with disruption of cerebrovascular and neuronal micro-environmental homeostasis (Tarumi and Zhang, 2018). We have demonstrated in a Phase1/2a clinical trial that transplantation of bone marrow mononuclear cell (BM-MNCs) after stroke accelerates functional recovery with improved cerebral circulation and metabolism (Taguchi et al., 2015). Furthermore, we have demonstrated that BM-MNC transplantation after stroke activates Hif-1α in endothelial cells via gap junction mediated cell–cell interaction followed by activation of angiogenesis and suppression of autophagy in murine model. Additionally, there is the transfer of low molecular weight substances from transplanted BM-MNCs to cerebral endothelial cells through gap junction, which is the major therapeutic mechanism of BM-MNC transplantation (Kikuchi-Taura et al., 2020). However, the link between BM-MNC transplantation and activation of cerebral metabolism was unclear.

Glucose is the major energy source for cerebral metabolism, and reduction in its transport had been shown in Alzheimer’s disease (Keaney and Campbell, 2015; Szablewski, 2017; Vallée et al., 2018). Although it is unclear whether this reduced glucose transport in Alzheimer’s disease is due to a lower energy demand or as a result of one of the primary insults, recent studies indicate that this is one of the causes of aggravation of the disease (Keaney and Campbell, 2015). Na^+^/K^+^-ATPases are responsible for the maintenance and restoration of ionic gradient across plasma membrane, and more than half of the ATP produced in the brain is used by Na^+^/K^+^-ATPases (Shrivastava et al., 2018). The aim of this study was to explore the potential link between BM-MNC transplantation and activation of cerebral metabolism using aged mice. We report herein that transplant of BM-MNCs in aged mice restores the neurological function by activating transcription of major energy supply and consumption related genes in the brain.

## Materials and Methods

All animal experiments were approved by the Animal Care and Use Committee of Foundation for Biomedical Research and Innovation at Kobe (protocol number: IBRI 18-05) and comply with the Guide for the Care and Use of Animals published by the Japanese Ministry of Education, Culture, Sports, Science and Technology. Experiments and results are reported according to the ARRIVE guidelines.

### BM-MNC Preparation

Bone marrow was obtained from 5-week-old syngeneic male CB-17 mice (Oriental Yeast, Tokyo, Japan). The femoral and tibial bones were dissected, distal ends were snipped using precision scissors, and BM was flushed out using PBS. BM was mechanically dissociated into single cells, and BM-MNCs were isolated by Ficoll-Paque (GE Healthcare, Little Chalfont, United Kingdom) density-gradient centrifugation as described elsewhere (Okinaka et al., 2019). Briefly, BM-MNCs were centrifuged at 400 × *g* for 40 min. Cells in buffy coats were isolated manually and washed three times with saline. It should be noted that BM-MNCs are not a single population (Nakano-Doi et al., 2010), and the significance of each population is under argument (Quadura et al., 2018). Freshly isolated cells were injected to mice without cell culture or expansion. Injection of BM-MNCs into aged mice was done to evaluate the change of neurological function.

**FIGURE 1:**
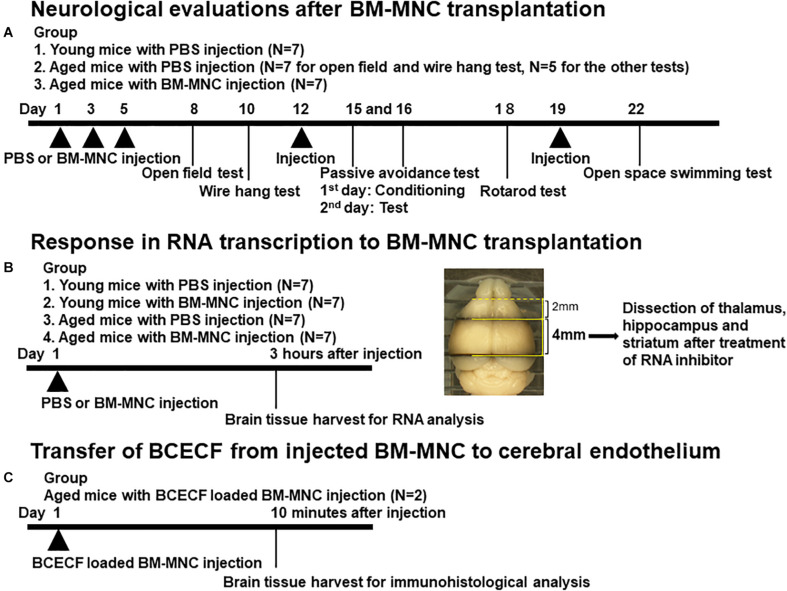
Design of *in vivo* experiments. **(A–C)** Schematic illustration of *in vivo* experimental design. The brain section used for RNA extraction was shown in **(B)**.

To evaluate the neurological function after BM-MNC transplantation in aged mice, male CB-17 mice aged more than 80 weeks were used, whereas 5-week-old-male CB-17 mice were used as young control group. Aged mice received 1 × 10^5^ BM-MNCs in 100 μl PBS (*N* = 7) or PBS alone (*N* = 7) by intravenous injection via tail veins (5 times in total). Young mice received 100 μl PBS (5 times in total) (*N* = 7). The reason for including healthy young was to provide information relating to the neurological and cognitive function in the steady state and thus confirm whether there has been some impairment in aged subjects. The major focus of this article was to investigate the restoration of impaired neurological function in aged mice by BM-MNC transplantation but not the improvement of neurological function in healthy young subjects. Therefore, no group for BM-MNC transplantation was prepared. Neurological tests were performed thereafter. Experimental design is shown in Figure 1A.

### Neurological Tests

Mice were evaluated using a panel of behavior tests, including open field test, wire hang test, passive avoidance test, rotarod test, and open space swimming test. For open field test, mice were allowed to search freely in a square acrylic box (30 × 30 cm) for 60 min. Lighting was kept on during the first 30 min (light period) and was subsequently turned off for 30 min (dark period). The total number of times animals crossed infrared beam [2 cm above the floor and spaced at 10 cm intervals both on *X*- and *Y*-axis (locomotion), and 5 cm above the floor spaced at 3 cm intervals on the *X*-axis (rearing)] was counted. For wire hang test, mice were placed on the metal wire grid (12 mm interval). The metal wire grid was then inverted and mouse was suspended at 20 cm height above the mouse cage and the latency to falls was recorded. Each mouse was tested five times with a 1 min interval between each trial, and the average time to fall was calculated. In the conditioning and acquisition phases of the passive avoidance tests, each mouse was placed in a white and illuminated compartment. Mice received a mild electric foot shock when crossing an adjacent dark compartment. At the next day, the mouse was again placed in the white compartment, and the time to cross over to the black compartment was measured as the test phase. For the rotarod test, each mouse was placed on a rotating lane rotating at 35 rounds per min and the time latency to fall from the rotarod was measured. Each mouse was tested three times with a 1 min recovery break between the trial, and average of the latency was employed. For open space swimming test, mice were placed in a swimming pool (200 cm diameter), and the distance of swimming for 600 s was recorded. All of the tests were conducted and analyzed by blinded investigators. Experimental design with the date of each behavior test is shown in Figure 1A.

### Injection of BM-MNCs to Evaluate the Change of RNA Transcription

To evaluate the change in RNA transcription by BM-MNCs in aged (more than 80 weeks) and young (5 weeks) mice, 1 × 10^5^ BM-MNCs in 100 μl PBS or PBS alone were intravenously injected via tail veins (one time), and response to BM-MNC injection was evaluated at 3 h after injection (*N* = 7 in each group). To investigate the characteristics of the reaction to BM-MNC transplantation in aged mice, young mice with BM-MNC transplantation were also prepared in this study. Experimental design is shown in Figure 1B.

### Quantitative PCR Analysis

Brain tissue was harvested at 3 h after transplantation of BM-MNCs or PBS. After harvesting the brain, coronal sections (4 mm thick) of the forebrain between 2 mm and 6 mm from frontal pole (Figure 1B) were cut followed by immersion in RNAlater (Thermo Fisher, Waltham, MA, United States) to prevent RNA degradation. Under stereoscopic microscope (Olympus, Tokyo, Japan), the part of thalamus, hippocampus, and striatum was dissected by medical tweezers, as described previously (Spijiker, 2011). Total RNA was isolated using RNeasy Plus Universal Mini Kit (Qiagen, CA, United States) according to manufacturer’s instructions. cDNA was synthesized from 1 μg total RNA using PrimeScript^TM^ II 1st strand cDNA Synthesis Kit (TAKARA, Kyoto, Japan) according to manufacturer’s protocol. Transcription of mRNA at hippocampus, striatum, and thalamus was analyzed using PowerUp^TM^ SYBR^TM^ Green Master Mix (Applied Biosystems, Foster City, CA, United States) and the Agilent AriaMx real-time quantitative PCR System. 18S ribosomal RNA was used for the reference gene. The list of target genes, primer sequences, and amplification protocols are shown in Table 1.

**TABLE 1:** Target genes, primer list and amplification protocol (mouse).

Gene	NCBI Accession No.	Sequences		
m18S	NR_003278.3	Forward	ACTCAACACGGGAAACCTCACC	
		Reverse	CCAGACAAATCGCTCCACCA	
mGlut1	NM_011400.3	Forward	TGGCGGGAGACGCATAGTTA	
		Reverse	CTCCCACAGCCAACATGAGG	
mGlut3	NM_011401.4	Forward	GAGGAACACTTGCTGCCGAG	
		Reverse	CTGGAAAGAGCCGATCGTGG	
mGlut4	NM_009204.2	Forward	CTTATTGCAGCGCCTGAGTC	
		Reverse	GGGTTCCCCATCGTCAGAG	
mMCT1	NM_009196.4	Forward	AGTGCAACGACCAGTGAAG	
		Reverse	GCGATCATTACTGGACGGCT	
mMCT2	NM_001358496.1	Forward	CAGCAACAGCGTGATAGAGC	
		Reverse	AGGCTGGTTGCAGGTTGAAT	
mMCT4	NM_001038653.1	Forward	GGCTGGCGGTAACAGAGTA	
		Reverse	CGGCCTCGGACCTGAGTATT	
mNa+/K+-ATPaseα1	NM_144900.2	Forward	TATCCTACTGCCCCGGGATG	
		Reverse	CTTCCGCACCTCGTCATACA	
mNa+/K+-ATPaseα2	NM_178405.3	Forward	TTCGCTAGCATCGTGGTTGT	
		Reverse	CAGCCAAAGCCGTCTCTTCT	
mNa+/K+-ATPaseα3	NM_001290469.1	Forward	TTGCCAAGGGTGTGGGTATC	
		Reverse	GGTGAAGTCCTTGAGGTCGG	
mHif1α	NM_001313919.1	Forward	AGCCAGCAAGTCCTTCTGAT	
		Reverse	AGGCTGGGAAAAGTTAGGAGTG	
mPHD3	NM_028133.2	Forward	ATCCACATGAAGTCCAGCCC	
		Reverse	ACACCACAGTCAGTCTTTAGCA	
mPDK1	NM_172665.5	Forward	TGCAAAGTTGGTATATCCAAAGCC	
		Reverse	TGTGCCGGTTTCTGATCCTT	

**Segment**	**Plateau**	**Temperature**	**Duration**	**Cycle**

Hot Start	1	50	0:03:00	1
Hot Start 2	1	95	0:03:00	1
Amplification	1	95	0:00:05	40
Amplification	2	60	0:00:30	40
Melt	1	95	0:00:30	1
Melt	2	65	0:00:30	1
Melt	3	95	0:00:30	1

### *In vivo* Transfer of Low Molecular Weight Fluorescence Molecular in Cytoplasm of BM-MNCs to Cerebral Endothelium

Bone marrow mononuclear cells were incubated with 5 μM of BCECF-AM (2′,7′-bis-(2-carboxyethyl)-5-(and-6)-carboxyfluorescein, acetoxymethyl ester, Dojindo, Kumamoto Japan) for 30 min at 37°C according to the manufacturer’s protocol. BCECF-AM was converted to BCECF (2′,7′-bis-(2-carboxyethyl)-5-(6)-carboxyfluorescein) at cytoplasm, and BCECF-loaded BM-MNCs were washed twice with PBS before injection. 1 × 10^5^ BCECF-loaded BM-MNCs in 100 μl PBS was injected to aged mice (80 weeks old, *N* = 2), and the transfer of BCECF from transplanted BM-MNCs to cerebral endothelium was evaluated by immunohistochemistry as described previously (Kikuchi-Taura et al., 2020). Briefly, at 10 min after cell injection, mice were anesthetized with sodium pentobarbital. The brain was removed, fixed with 4% paraformaldehyde (PFA; Thermo Fisher), and cut into coronal sections (20 μm) using a vibratome (Leica, Wetzlar, Germany). Sections were immunostained with primary antibodies against CD31 (BD Pharmingen, San Jose, CA, United States; dilution 1:50) and DAPI (Thermo Fisher, 1:1,000). Alexa 555 (Novus Biologicals, Centennial, CO, United States)-coupled antibody was used as the secondary antibody to visualize CD31 antibody. Confocal images were obtained with confocal microscope (Fluoview FV1000: Olympus). Experimental design is shown in Figure 1C.

### Co-culture of Mouse-BM-MNCs and Human Umbilical Vein Endothelial Cells (HUVEC)

Human umbilical vein endothelial cells (Kurabo, Osaka, Japan) were cultured with medium, serum, and growth factors (HuMedia-EB2, Kurabo) according to the manufacturer’s protocol. HUVEC in passage 6 were used for co-culture with mouse BM-MNCs. To evaluate the effect of BM-MNCs on RNA transcription in HUVEC *in vitro*, HUVEC were suspended in the 150 μl medium (2 × 10^5^ cells/tube) and incubated for 3 h with mouse BM-MNCs (2 × 10^6^ cells suspended in 20 μl PBS) at 37 degree with 5% CO_2_. Same number and volume of HUVEC added with 20 μl PBS at the same condition was prepared as the control. Total RNA extraction and cDNA synthesize were performed as we described above. The changes in transcription in HUVEC were evaluated by qPCR. The list of target genes, primer sequences, and amplification protocols is shown in Table 2. It was confirmed for all human primers that no signal was detected by amplification of mouse cDNA.

**TABLE 2:** Target genes, primer list, and amplification protocol (human).

Gene	NCBI Accession No.	Sequences		
hTBP	NM_003194.5	Forward	CGCCGGCTGTTTAACTTCG	
		Reverse	TGGGTTATCTTCACACGCCA	
hHif1α	NM_001530.3	Forward	CCAGACGATCATGCAGCTACT	
		Reverse	TGATTGCCCCAGCAGTCTAC	
hPHD3	NM_022073.3	Forward	GATCGTAGGAACCCACACGA	
		Reverse	TCAGAGCACGGTCAGTCTTC	
hPDK1	NM_001278549.1	Forward	GCAAAATCACCAGGACAGCC	
		Reverse	TCTGTTGGCATGGTGTTCCA	
hGlut1	NM_006516.3	Forward	CCTGCAGTTTGGCTACAACAC	
		Reverse	CAGGATGCTCTCCCCATAGC	
hMCT1	NM_003051.3	Forward	GTACTGGAACAAGCAAACGAGG	
		Reverse	TCCAAAATGCAGGTCAAATCC	

**Segment**	**Plateau**	**Temperature**	**Duration**	**Cycle**

Hot Start	1	50	0:03:00	1
Hot Start 2	1	95	0:03:00	1
Amplification	1	95	0:00:05	40
Amplification	2	60	0:00:30	40
Melt	1	95	0:00:30	1
Melt	2	65	0:00:30	1
Melt	3	95	0:00:30	1

### Statistics

Statistical comparisons among groups were determined using one-way analysis of variance (ANOVA) followed by *post hoc* analysis using Dunnett’s test. Where indicated, individual comparisons were performed using Student’s *t*-test. All data are shown as mean ± SD.

## Results

### BM-MNC Transplantation Restored Neurological Functions of Aged Mice

**FIGURE 2:**
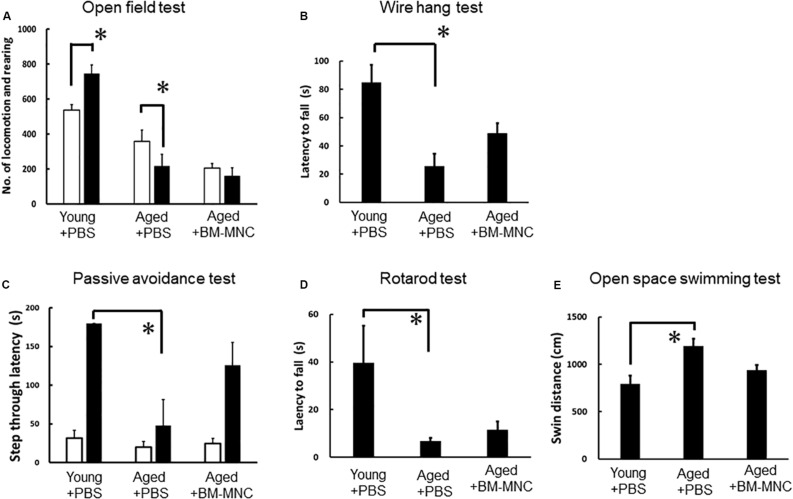
Effect of BM-MNC transplantation on neurological functions of aged mice. **(A)** Paradoxical decrease of locomotion/rearing counts in response to darkness in aged mice was released by BM-MNC transplantation. **(B)** Significant impairment of motor function in aged mice was restored by BM-MNC transplantation. **(C)** Significant impairment of learning/memory in aged mice was restored by BM-MNC transplantation. **(D)** Significant impairment of coordinated motion/motor learning in aged mice was restored by BM-MNC transplantation. **(E)** Excess swimming in aged mice was released by BM-MNC transplantation. White or black bars indicate the number of locomotion/rearing in light or dark condition, respectively. *p* < 0.05 versus light condition. **(A)** *p < 0.05 vs young mice injected with PBS **(B–E)**. *N* = 7 in each group **(A,B)**. *N* = 7 in young and aged mice with BM-MNC transplantation, *N* = 5 in aged mice injected PBS [**(C–E)**, two mice were euthanized before passive avoidance test due to the body weight loss according to predetermined protocol for animal experiment].

To evaluate the effect of BM-MNC transplantation, neurological functions in aged mice with BM-MNC transplantation were compared with those in aged and young mice injected with PBS. In the open field test, young mice injected with PBS showed increased activity in response to dark condition as expected. In contrast, aged mice injected with PBS showed reduced activity in response to dark condition. Aged mice with BM-MNC transplantation showed no significant increased/reduced activity in response to dark condition (Figure 2A). Neurological analysis by wire hang test revealed a significant reduction of the latency to fall in aged mice injected with PBS, compared with young mice. In contrast, there was no significant difference in the latency in aged mice injected with BM-MNCs, compared with young mice (Figure 2B). The passive avoidance test revealed an impaired learning and memory in aged mice injected with PBS, compared with young mice. In contrast, there was no significant difference between aged mice injected with BM-MNCs and young mice injected with PBS (Figure 2C). The rotarod test revealed a decreased latency to falling in aged mice injected with PBS, although no significant difference was observed in mice that received BM-MNC transplantation compared with young mice (Figure 2D). Compared with young mice, open space swimming test revealed increased swim response in aged mice injected with PBS, although no significant difference was observed in mice injected with BM-MNCs (Figure 2E).

### Change in Transcription of Glucose Transporter by BM-MNC Transplantation

**FIGURE 3:**
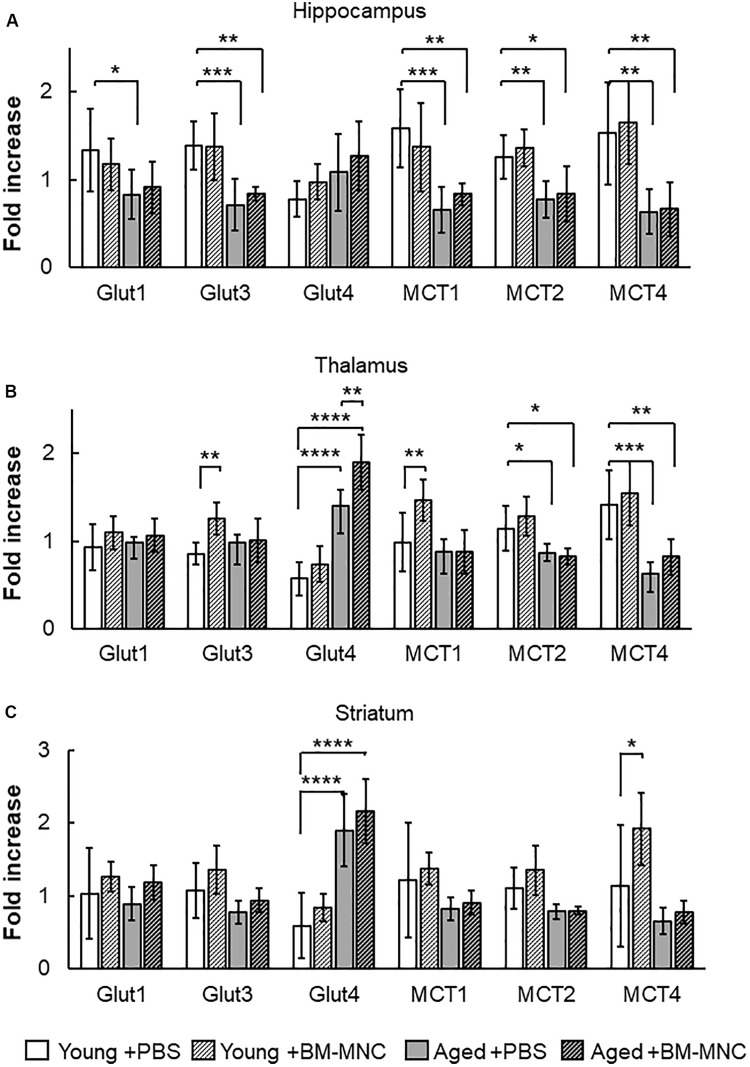
Response in RNA transcription of energy source transporters to BM-MNC transplantation. **(A)** In the hippocampus, significant reduction of Glut1 in aged mice was restored by BM-MNC transplantation. The transcription of Glut3, MCT1, MCT2, and MCT4 significantly decreased in aged mice with and without BM-MNC transplantation, compared with young mice with PBS injection. **(B)** In the thalamus, transcription of Glut4 significantly increased in aged mice, compared with young mice with PBS injection. BM-MNC transplantation significantly increased Glut4 transcription in aged mice. BM-MNC transplantation significantly increased Glut3 and MCT1 transcription in young mice. **(C)** In the striatum, transcription of Glut4 significantly increased in aged mice with and without BM-MNC transplantation. BM-MNC transplantation significantly increased MCT4 transcription in young mice. *N* = 7, each. *p < 0.05, **p < 0.01, ***p < 0.001, ****p < 0.0001. *p*-values in comparison between young mice with BM-MNC injection and aged mice with PBS or BM-MNCs were not labeled to avoid too much complexity **(A–C)**.

An increase in cerebral metabolism had been shown to occur in stroke patients after BM-MNC transplantation (Taguchi et al., 2015). To investigate the effects of BM-MNC transplantation on cerebral metabolism in aged mice, the transcription of energy source transporters, including glucose transporters and monocarboxylate transporters, was investigated by qPCR. In the hippocampus, the transcription of glucose transporter 1 (Glut1), Glut3, monocarboxylate transporter 1 (MCT1), MCT2, and MCT4 was shown to be significantly decreased in aged mice injected with PBS, compared with young mice injected with PBS (Figure 3A). It was noteworthy that the significant difference in the transcription of Glut1 between aged and young mice was absent in the case of BM-MNC transplantation. In the thalamus, a significantly reduced transcription of MCT2 and MCT4 was observed in aged mice, compared with young mice (Figure 3B). In contrast, increased transcription of Glut4 was observed in aged mice, compared with young mice, and the transcription of Glut4 was significantly higher in aged mice with BM-MNCs, when compared with aged mice with PBS. In the striatum, significant increased transcription of Glut4 was observed in aged mice, compared with young mice (Figure 3C). It was noteworthy that transcription of Glut4 was significantly increased in aged mice, compared with young mice at thalamus and striatum.

### Change in Transcription of Na^+^/K^+^-ATPase Following BM-MNC Transplantation

**FIGURE 4:**
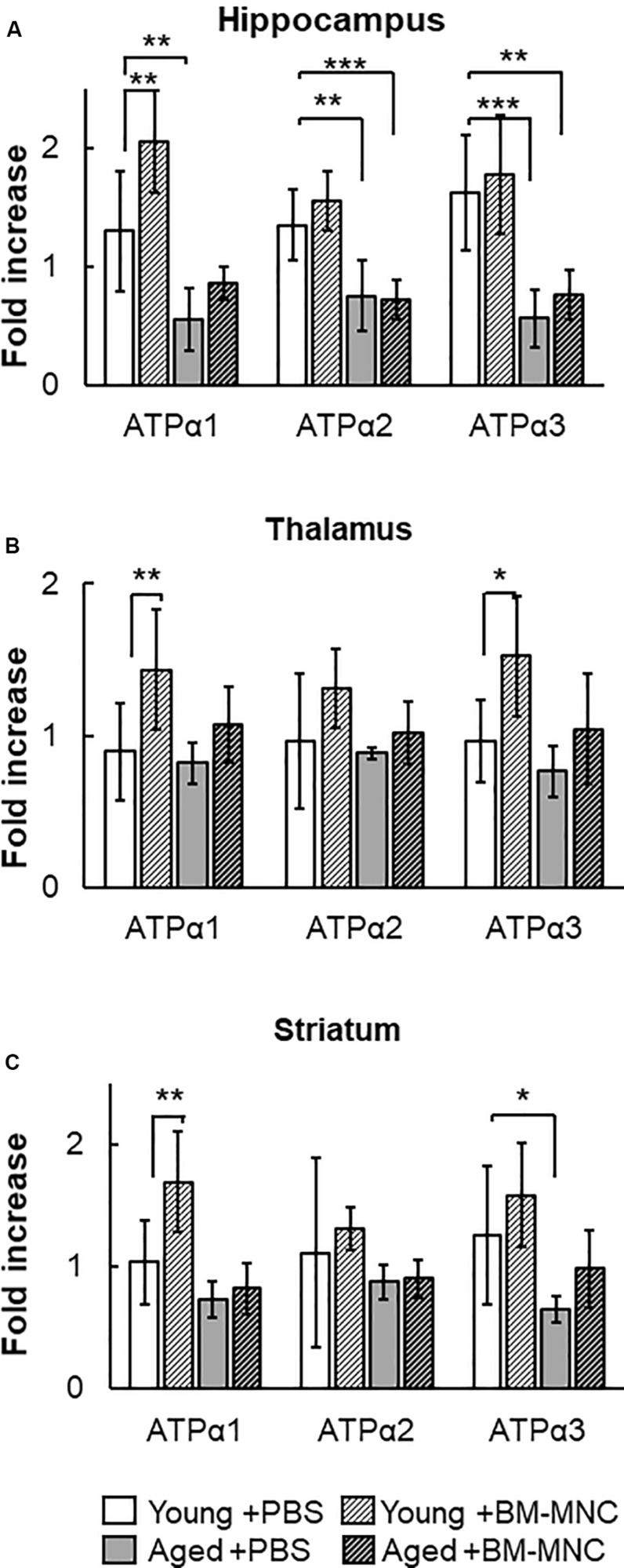
Response in RNA transcription of Na^+^/K^+^-ATPase to BM-MNC transplantation. **(A)** In the hippocampus, significant reduction of Na^+^/K^+^-ATPase α1 in aged mice was restored by BM-MNC transplantation. The transcription of ATPase α2 and α3 significantly decreased in aged mice with and without BM-MNC transplantation, compared with young mice. **(B)** BM-MNC transplantation significantly increased ATPase α1 and α2 transcription in young mice. **(C)** Significant reduction of Na^+^/K^+^-ATPase α3 transcription in aged mice was restored by BM-MNC transplantation at striatum. BM-MNC transplantation significantly increased ATPase α2 transcription in young mice. *N* = 7, each. *p < 0.05; **p < 0.01; ***p < 0.001. *p*-values in comparison between young mice with BM-MNC injection and aged mice with PBS or BM-MNCs were not labeled to avoid too much complexity **(A–C)**.

The Na^+^/K^+^-ATPase is an ATP-consuming system in the central nervous system (Howarth et al., 2012). The change in the transcription of Na^+^/K^+^-ATPase was investigated in mice undergoing BM-MNC transplantation. In the hippocampus, the transcription of Na^+^/K^+^-ATPase α1 was significantly reduced in aged mice injected with PBS, compared with young mice (Figure 4A). However, significant difference was absent following BM-MNC transplantation, and BM-MNC transplantation increased Na^+^/K^+^-ATPase α1 transcription in young mice. The transcription of Na^+^/K^+^-ATPase α2 and α3 was significantly reduced in aged mice injected with PBS or BM-MNC transplantation, compared with young mice. In contrast, there were no significant differences in the transcription of Na^+^/K^+^-ATPase α1, α2, and α3 in the thalamus, although BM-MNC transplantation increased Na^+^/K^+^-ATPase α1 and α3 transcription in young mice (Figure 4B). When comparing the striatum in young mice, transcription of Na^+^/K^+^-ATPase α3 was reduced in aged mice injected with PBS, although no significant difference was observed in aged mice, which underwent BM-MNC transplantation (Figure 4C). BM-MNC transplantation increased Na^+^/K^+^-ATPase α1 transcription in young mice.

### Change in Transcription of Hif-1α Following BM-MNC Transplantation in Aged Mice

**FIGURE 5:**
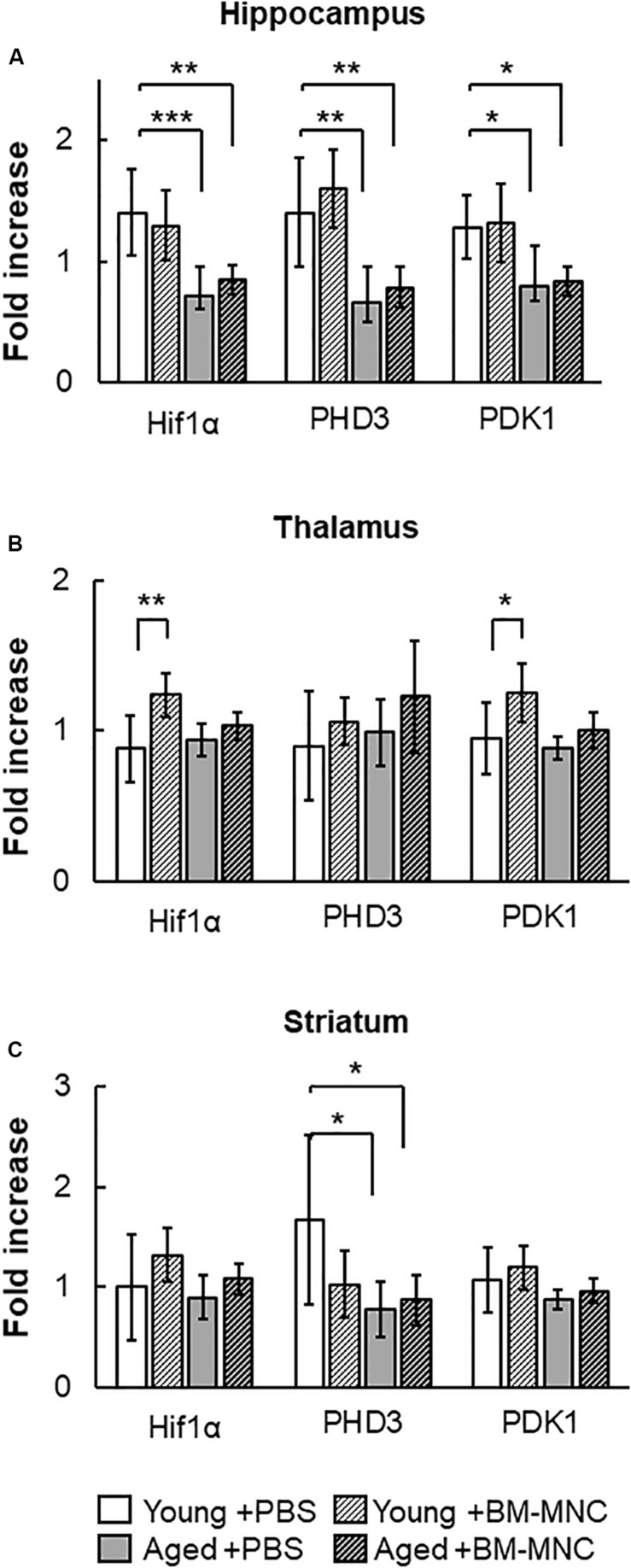
Response in RNA transcription of Hif-1α, PHD3 and PDK1 to BM-MNC transplantation. **(A)** In the hippocampus, transcription of Hif-1α, *PHD3*, and *PDK1* significantly decreased in aged mice with and without BM-MNC transplantation, compared with young mice with PBS. **(B)** BM-MNC transplantation significantly increased Hif-1α and PDK transcription in young mice. **(C)** In the striatum, transcription of PHD3 significantly decreased in aged mice with and without BM-MNC transplantation, compared with young mice with PBS. *N* = 7, each. *p < 0.05; **p < 0.01; ***p < 0.001. *p*-values in comparison between young mice with BM-MNC injection and aged mice with PBS or BM-MNCs were not labeled to avoid too much complexity **(A–C)**.

Hif-1α is one of the major transcriptional factors that regulates the expression of energy source transporters (Zorzano et al., 2005; Masoud and Li, 2015). To investigate the effect of BM-MNC transplantation in aged mice, the transcription of Hif-1α and its down-stream genes, *PHD3* and *PDK1*, were investigated. As shown in Figure 5A, reduced expression of Hif-1α, *PHD3* and *PDK1* was observed in the hippocampus of aged mice, compared to young mice. No differences were observed in the thalamus between groups of mice (Figure 5B), and reduced expression of PHD3 was observed at striatum in aged mice, compared with young mice (Figure 5C). In young mice, BM-MNC transplantation increased Hif-1α and *PDK1* transcription in the thalamus.

### *In vivo* Transfer of Low Molecular Weight Substance From BM-MNCs to Endothelial Cells

**FIGURE 6:**
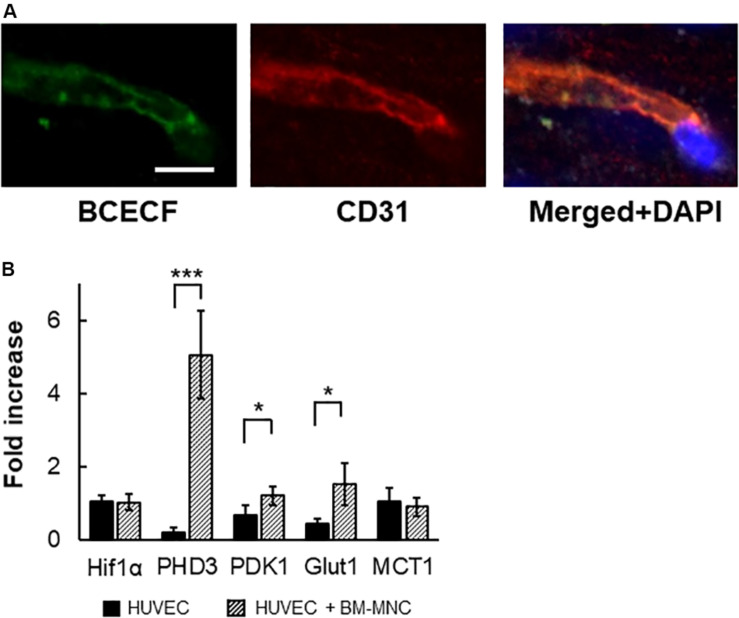
*In vivo* transfer of low molecular weight substance from BM-MNCs to endothelial cells. **(A)** Representative picture at 10 min after BCECF-loaded BM-MNC transplantation at hippocampus. Transferred BCECF from transplanted BM-MNCs was observed in endothelial cells. **(B)** Increased transcription of PHD3, PDK1 and Glut1 in endothelial cells by BM-MNCs *in vitro*. Significant increase in transcription of *PHD3*, *PDK1*, and Glut1 in HUVEC were observed when co-cultured with BM-MNCs. No significant change in transcription of Hif-1α and MCT1 was observed. Scale bar: 10 μm **(A)**. *N* = 4, each, *p < 0.05; ***p < 0.001 **(B)**.

We had shown that transfer of low molecular weight substance from transplanted BM-MNCs to cerebral endothelial cells is the prominent pathway in the activation of endothelial cells by BM-MNC transplantation after cerebral ischemia (Kikuchi-Taura et al., 2020). To evaluate the transfer of low molecular weight substance from transplanted BM-MNCs to cerebral endothelial cells in aged mice, BCECF-loaded BM-MNCs were injected, and mice were sacrificed at 10 min after injection. As shown in Figure 6A, BCECF-positive endothelial cells were observed in the hippocampus in aged mice.

### Change in Transcription in HUVEC by Co-culture With Mouse BM-MNCs

To investigate the effect of BM-MNCs on endothelial cells, HUVEC were co-cultured with mouse BM-MNCs, and the change in RNA transcription was investigated. Although no significant differences were observed in the transcription of Hif-1α, a significant increase in the transcription of *PHD3* and *PDK1* was observed (Figure 6B). Furthermore, increased transcription of Glut1, but not MCT1, was observed in the co-culture with BM-MNCs.

## Discussion

### Resource Identification Initiative

We demonstrate herein that intravenous BM-MNC transplantation in aged mice has the potential to restore a series of age-related neurological impairments, which are related to increased transcription of glucose transporter and Na^+^/K^+^-ATPase in the hippocampus.

Cerebral metabolism is known to decrease both in normal aging and Alzheimer’s disease, and a correlation between decreased metabolism and neurological impairments has been reported (Mosconi, 2013). Consistent with these findings, a significant decrease of glucose/monocarboxylate transporter transcription, including Glut1, Glut3, MCT1, MCT2, and MCT4, has been observed in the hippocampus in aged mice with impaired neurological function. The Na^+^/K^+^-ATPase consumes the most ATP in the central nervous system and is essential for its function. A significant decrease in the transcription of glucose/monocarboxylate transporters (except Glut4), Na^+^/K^+^-ATPase α1, α2, and α3 was observed in aged mice which indicate that the restoration of their expression may facilitate the re-establishment cerebral metabolism and neuronal activity. To this end, BM-MNC transplantation after stroke has been shown to improve cerebral circulation and metabolism with improved functional recovery (Nakagomi et al., 2009; Taguchi et al., 2015). In the study reported herein, we demonstrate that BM-MNC transplantation in aged mice improves a series of age-related neurological impairments which result from increased transcription of Glut1 and Na^+^/K^+^-ATPase α1 in the hippocampus. Compared with the hippocampus, milder differences in RNA transcription between young and aged mice were observed in the thalamus and the striatum. As the characteristic of hippocampus, neurogenesis is known to occur primarily in hippocampus and the subventricular zone of the lateral ventricles with an age-dependent decline (Takei, 2019). Furthermore, blood–brain barrier breakdown in the aging hippocampus had been reported (Montagne et al., 2015). Our results were consistent with these previous reports that hippocampus is vulnerable to age-related insults.

Recently, we had shown that transfer of low molecular weight substance, including glucose, from transplanted BM-MNCs to cerebral endothelial cells, is the prominent pathway in the activation of endothelial cells by BM-MNC transplantation after cerebral ischemia, and the transfer of low molecular weight substance suppresses autophagy in injured endothelia cells (Kikuchi-Taura et al., 2020). In this article, transfer of low molecular weight substance from transplanted BM-MNCs to cerebral endothelial cells was observed in non-stroke aged mice within 10 min after BM-MNC transplantation. The therapeutic mechanism by which BM-MNC transplantation improves neurological functions is now partially resolved and is related to an improvement in cerebral metabolism with increased transcription of Glut1 and neurological functions accompanied by increased transcription of Na^+^/K^+^-ATPase α1. Quantification of these transcriptions could also be used as surrogate biomarkers for age-related neurological impairment and evaluating the efficacy of therapies. It is notable that BM-MNC transplantation in young mice increased the expression of Na^+^/K^+^-ATPase α1 in the hippocampus, thalamus, and striatum, and further studies are required to reveal the link between increased the expression of Na^+^/K^+^-ATPase α1 and change of behavior in young mice after BM-MNC transplantation.

Glut4 is insulin responsive and the primary glucose transporter in skeletal muscle, heart, and adipose tissue (Reno et al., 2017). Glut4 is also expressed in neuron at brain and suggested to play a role in providing additional glucose to neurons under conditions of additional energy demand (Apelt et al., 1999). In contrast to reduced transcription of Glut1 and Glut3 in the hippocampi of aged mice, no reduction at hippocampus and significant increase at striatum and thalamus of Glut4 was observed in aged mice, compared with young mice. These findings may be explained by the reduced expression of Hif-1α at hippocampus, which is the strong activator of Glut1 and 3, but not Glut4 (Zorzano et al., 2005; Masoud and Li, 2015). Furthermore, the increased transcription of Glut4 in the striatum and thalamus would indicate an increased energy demand caused by decreased transcription of MCTs in aged mice, where lactate is one of the most important energy sources of neurons (Bélanger et al., 2011).

By co-culture with BM-MNCs, increased transcription of *PHD3*, *PDK1*, and Glut1 was observed in HUVECs *in vitro*. The activation of Hif-1α is regulated by post-translational modifications (Ke and Costa, 2006), and the transcription of *PHD3* and *PDK1* is known to be increased after activation of Hif-1α (Ognibene et al., 2017). Activation of Hif-1α is also known to upregulate Glut1 transcription (Lokmic et al., 2012). These findings are consistent with our recent report that transplanted BM-MNCs activate Hif-1α at endothelial cells via gap junction mediated cell–cell interaction in murine stroke model (Kikuchi-Taura et al., 2020). Similar to BM-MNC transplantation in murine stroke model, transfer of low molecular weight substance from transplanted BM-MNCs to cerebral endothelial cells was observed in aged mice. Our *in vitro* and *in vivo* data indicated that activation of Hif-1α cascade by BM-MNCs would be one of the triggers that upregulate Glut1 transcription at endothelial cells.

Despite the above, our study does have limitations. First, the change of actual metabolic substances or protein level after BM-MNC transplantation was not measured, although the increase of cerebral metabolism had been reported in clinical trial of BM-MNC transplantation for stroke at 6 months after cell transplantation (Taguchi et al., 2015). Similar to a previous report of BM-MNC transplantation in stroke mice (Kikuchi-Taura et al., 2020), transfer of low molecular weight molecules from BM-MNCs is observed in cerebral endothelial cells in aged brain within 10 min after cell injection and transplanted BM-MNCs were no longer observed. Furthermore, we had previously shown that transplantation of hematopoietic stem cell, one of the cell populations of BM-MNCs, after stroke activates cerebral vasculature within 24 h after cell injection followed by neurological recovery up to 90 days (Taguchi et al., 2015). Further studies are required to elucidate the mechanism of activation of cerebral vasculature after cell injection and the neurological changes in the later periods. This should include investigating the subsequent changes in RNA transcription, protein synthesis, its maturation/activation/inactivation and appropriate cellular localization followed by changes in the level of metabolic substrates. However, the change of RNA transcription can directly reflect the response of brain tissue against BM-MNC transplantation and would be able to explain the clinical finding in stroke patients that showed increased cerebral metabolism after BM-MNC transplantation. Although the change in RNA transcriptions by BM-MNC transplantation was not drastic, a trend that BM-MNC transplantation restored many of transcription of energy source transporters and Na^+^/K^+^-ATPases was observed at hippocampus. This information can provide working hypotheses for further studies aiming to restore age-related impairments by activation of energy sources transfer to brain tissue. Second, experiments were only performed in male animals. Hence, we were unable to detect any potential gender effect. However, it must be noted that this study was an exploratory, not a confirmative one. Enrolling a mixed-sex population would have meant to double animal numbers in anticipation of a gender effect. Any gender effects are necessary in downstream confirmative research to complete our understanding on BM-MNC effects in aged subjects.

## Conclusion

In conclusion, our results demonstrated that the transcription of Glut1, Glut3, MCT1, MCT2, and MCT4 at hippocampus is decreased with neurological impairments in aged mice, and transplantation of BM-MNCs restored the transcription of Glut1 and Na^+^/K^+^-ATPase α1 with neurological functional recovery. Our data also indicated that the level of Glut1 and Na^+^/K^+^-ATPase α1 at hippocampus can be the surrogate markers of functional recovery in aged mice after cell transplantation.

## Data Availability Statement

All datasets generated for this study are included in the article/supplementary material.

## Ethics Statement

The animal study was reviewed and approved by Animal Care and Use Committee of Foundation for Biomedical Research and Innovation at Kobe.

## Author Contributions

YT performed the qPCR analysis. YOk transplanted cells. YOg and AK-T did behavior analysis. JB and AT designed the study. AT wrote the manuscript. SG, CC, and YK supervised the project and revised the manuscript critically for important intellectual content.

## Conflict of Interest

The authors declare that the research was conducted in the absence of any commercial or financial relationships that could be construed as a potential conflict of interest.
